# Appropriateness of Head CT Scans at Tikur Anbessa Specialized Hospital, Ethiopia

**DOI:** 10.4314/ejhs.v32i2.17

**Published:** 2022-03

**Authors:** Etsehiwot Demeke, Abebe Mekonnen

**Affiliations:** 1 Department of Radiology, College of Health sciences, Addis Ababa University, Addis Ababa, Ethiopia

**Keywords:** Overutilization, Unnecessary procedures, Computed tomography, neuroimaging

## Abstract

**Background:**

Overutilization of advanced diagnostic imaging modalities strains health care systems, especially in resource limited setups. The aim of this study is to identify magnitude of inappropriate Head Computed Tomography scans at Tikur Anbessa Specialized Hospital.

**Methods:**

Retrospective cross-sectional study was conducted at Tikur Anbessa Specialized Hospital, Radiology department, among patients getting Head Computed Tomography examinations in the period of August 2018- November 2018. Appropriateness of each scan was assessed using the American College of Radiology Appropriateness Criteria.

**Result:**

Of the 443 Head Computed Tomography scans assessed, 61.6% were done for male patients and the mean age of patients scanned is 35. Children younger than 14yrs of age constituted 17.2%. No contrast was used in 63.9% of the scans and 64.3% were initial imaging with no prior study for similar indication. Out of the scans evaluated, 11.7% were inappropriate. Headache (38.5%), Seizure (23.1%) and Head trauma (23.1%) were the commonest indications for inappropriate scan. Scans done for cerebrovascular disease were 240 times more likely to be appropriate. Large number of inappropriate scans were requested from central triage (33.3%) and adult emergency (26.2%). Pediatric department requested inapproprieate scans in 11.9% of the cases. Residents requested majority of inappropriate scans (82.3%). Inappropriateness was associated with use of contrast agent and having only incidental outcomes.

**Conclusion:**

A large number of inappropriate Head Computed Tomography scans are being done. Mechanisms such as preauthorization by radiologists, increasing awareness by medical students, physicians, radiology residents and radiologists and preparing customized imaging appropriateness guidelines should be implemented.

## Introduction

Use of advanced diagnostic imaging has been growing significantly over the past couple of decades (1). The wide spread acceptance of these imaging modalities among patients and clinicians is due to the quick and easy clinical information they provide. A substantial fraction of the growth, however, is attributed to overutilization. Overutilization is defined as applications of procedures where circumstances indicate that they are unlikely to improve patient outcome(2). Prevalent use of imaging does not always improve health care quality, rather it puts a strain on the healthcare system (2,3).

The economical strain of overutilization is marked not only in resource limited setups but also in the western world(4, 5). In most African countries including Ethiopia, where health care expenditure is over reliant on out of pocket payment from patients, the financial burden directly relies on individual household (6, 7). Long waitlist in overburdened radiology departments and the radiation dose associated with CT examinations which is associated with carcinogenic potential, predominantly in children, is another issue (3, 8, 9). Widespread use of contrast media is also a growing concern, as it is associated with severe reactions as well as allergies and anaphylactic responses (10, 11).

This dramatic increment in rate of overutilization has motivated health systems worldwide to implement control mechanisms aimed at appropriate utilization of imaging examinations (1, 12, 13). Determining the appropriateness of medical imaging procedures is a complex task. Appropriateness may vary with the age, gender, size, and physical limitations of the patient and the symptoms being investigated (14). The clinical indication, the type of examination, the outcome of the scan, the use of contrast agents and whether there is a previous scan or not are other factors that need to be taken into consideration.

In 1993, the American college of radiology (ACR) introduced evidence-based guidelines to assist in making the most appropriate imaging for a specific clinical condition. These guidelines are revised annually. As of 2018, there are 178 diagnostic imaging and interventional radiology topics with over 912 variants of clinical scenarios for which appropriateness rating was devised (15).

Different Computer-based decision support programs are designed incorporating such guidelines (e.g. ACR Select) as a clinical decision support tool (16). Integrating clinical decision support tools for imaging requests resulted in increased overall appropriateness criteria scores(17).

In Ethiopia, CT service is not widely available in all the public hospitals. The few institutions equipped are overloaded with a large number of patients referred from all corners of the country. The number of radiologists in the country is also limited which resulted in long waitlists to get these examinations done as well as get the radiologic reports timely. This hindered patients from getting prompt clinical management and subjected them to unnecessary cost and exhaustion from choosing private vendors as an alternative to get treated faster. It is crucial to determine the magnitude of appropriateness of imaging and the associated contributing factors in order to forward possible strategies to provide better quality care and to efficiently utilize the limited resource available.

## Materials and Methods

A hospital-based retrospective cross-sectional study was done at Tikur Anbessa specialized hospital, Radiology department, by analyzing data collected from radiology requests of patients getting head CT examinations from August 2018 to November 2018.

All patients getting at least one head CT examination during the study period, with available request and complete/near-complete set of the required information were included. Scans with lost requests, request completeness of <80% and for whom medical records couldn't be retrieved, duplicated requests, CT scans done with paranasal sinus protocol, high-resolution temporal bone CT scans, head and Neck CT scans were excluded from the study.

The sample size was calculated before beginning the study, assuming an appropriateness rate of 50%, a margin of error of 5% and a 95% confidence level. Consequently, we sought to obtain a sample of 385 CT examinations. Anticipating an unavailability of clinical documentation in 15% of cases; the final sample size required was calculated to be 443 CT examinations. The sampling technique was convenience sampling during the study period until the required sample size is reached.

Data regarding demographics (sex and age), Medical Record Number (MRN), type of scan, use of contrast, indication for scanning, qualification of requesting physician, requesting department, mode of scan (urgent or elective), presence of previous CT or MRI, pre-evaluation by radiology resident/radiologist, outcome of the scan and whether the diagnostic hypothesis was confirmed or not, were recorded using a structured questioner. Medical records were reviewed for those requests which had incomplete information.

According to ACR-AC, appropriateness is rated on an ordinal scale that uses integers from 1 to 9 grouped into three categories: 1, 2, or 3 are in the category “Usually not appropriate”, where the harms of doing the procedure or treatment outweigh the benefits; and 7, 8, or 9 are in the category “Usually appropriate” where the benefits of doing a procedure or treatment outweigh the harms or risks.

The middle category is called “Maybe appropriate” and is represented by 4, 5, or 6 on the scale. The middle category describes when the risks and benefits are equivocal or unclear, the dispersion of the individual ratings from the panel rating is too large, the evidence is contradictory or unclear, or there are special circumstances or subpopulations which could influence the risks or benefits that are embedded in the variant.

The primary considerations when ACR devised the appropriateness ratings were the diagnostic utility, accuracy and test performance. The expert panel assumed that we are practicing in an ideal world where every procedure in the variant table is available and accessible disregarding the cost. It is also assumed that the patient does not have any contraindication for any of the procedures listed in the variant table and all procedures are performed and interpreted by an expert. The relative radiation level (RRL), radiation exposure and the radiation dose are not considered except when two procedures have nearly equivalent diagnostic accuracy or test performance.

The appropriateness of imaging was scored by the investigator, by referring to the respective tables for the specific clinical condition and variant in the ACR-AC. If a patient had received more than one diagnostic imaging examination during the study period, the judgment of appropriateness was carried out for each examination. Outcome of the scan was retrieved from the radiology reports in the PACS and were categorized into one of four categories; normal, incidental, abnormal finding affecting management and abnormal finding in a patient with a known diagnosis with no new finding. Scans with the following outcomes (normal, incidental and abnormal finding in a patient with a known diagnosis with no new finding) were conservatively considered as having the diagnostic hypothesis not confirmed. Whereas scans with abnormal finding affecting management were considered as having their diagnostic hypothesis confirmed. Data was entered and analyzed using SPSS software version 25.0. Data collection was commenced after ethical clearance was obtained from the department research ethical review committee. On the data collection form, anonymity was assured by omitting the names of patients.

## Results

**Figure 1:**
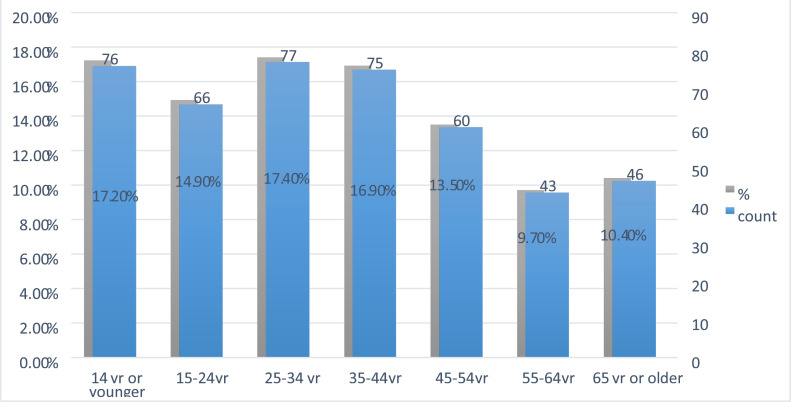
Age group distribution of patients who had head CT scans.

Of the 443 head CT scans, 170 (38.4) were done for female patients and 273 (61.6%) were done for male patients. The mean age of scanned patients was 35 (SD=20.8). Children aged 14 years or younger accounted for 76 (17.2 %) (Figure 1).

Overall, 2/3^rd^ of the scans did not use IV contrast and more than half of patients scanned had no prior cross-sectional imaging of the brain for similar illness (64.3%).

Some of the most common clinical indications for getting head CT included; head trauma (31.6%), headache (14.4%), cerebrovascular diseases (13.8%), post craniotomy control (12.6%) and acute mental status changes (10.2%). Hearing loss and vertigo, sinusitis, cranial neuropathy and ataxia are some of the least common reasons for requesting head CT.

Almost half of the scans were requested by interns (47.9%) while residents requested 39.5% of the scans. No scans were requested by consultants. The requesting physician was not mentioned in the request in 12.6% of scans. Only 30.2% of the requests were pre-evaluated by a radiology resident/ radiologist prior to scanning.

Adult Emergency Outpatient Department (EOPD) requested the highest number of scans (42%) followed by the neurosurgery department (11.3%) and the pediatric department requested 9.5% of the head CTs. Gynecology department requested the least number of scans (0.3%) (Table 1). Almost 3/4^th^ of the scans were done on an emergency basis.

**Table 1:** Distribution of appropriateness according to clinical indication of scan

Characteristic	total N (%)	Appropriate N (%)	May be appropriate N (%)	Inappropriate N (%)	Non-codable N (%)
Indication of scan					
Head Trauma	140 (31.6)	122 (87.1)	5 (3.6)	12 (8.6)	1 (0.7)
Acute mental status change	45 (10.2)	28 (62.2)	17 (37.8)	0 (0)	0 (0)
Cerebrovascular diseases	61 (13.8)	57 (93.4)	2 (3.3)	2 (3.3)	0 (0)
Headache	64 (14.4)	18 (28.1)	25 (39.1)	20 (31.3)	1 (1.6)
Seizure	23 (5.2)	4 (17.4)	5 (21.7)	12 (52.2)	2 (8.7)
Post craniotomy control	56 (12.6)	0 (0)	0 (0)	0 (0)	56 (100)
Others	54 (12.2)	1 (1.9)	13 (24.1)	6 (11.)	34 (63)

From the 443 brain CT scans, according to the ACR appropriateness criteria, 230 (51.9%) fell in the usually appropriate category whereas 15.1 % were in the ‘maybe appropriate’ category. Out of the scans evaluated, 52(11.7%) were deemed inappropriate. There were clinical indications which didn't fit into any of the ACR appropriratness criteria tables (21.3%). These were were assigned as ‘ACR non-codable’ (Figure 2). Majority of these scans are post craniotomy control CT scans (Table 1).

The highest number of inappropriate scans were requested from central triage (14 scans), followed by 11 scans from adult EOPD and 7 scans from internal medicine. Of the inappropriate scans, 11.9% of were requested from pediatric department and 26.9% of inappropriate scans were done for children 14 years or younger. Neurosurgery, Neurology, Oncology, and ICU were some of the departments requesting the least number of inappropriate scans (one scan from each) (Table 2).

**Table 2:** Distribution of appropriateness according to requesting department

Characteristic	Total	Appropriate	May be appropriate	Inappropriate	Non-codable
	N (%)	N (%)	N (%)	N (%)	N (%)
Requesting department					
Adult EOPD	186 (42)	159 (85.5)	10 (5.4)	11 (5.9)	6 (3.2)
Pediatrics	42 (9.5)	19 (45.2)	10 (23.8)	5 (1.9)	8 (19)
Internal medicine	30 (6.8)	10 (33.3)	13 (43.3)	7 (23.3)	0 (0)
Neurosurgery	50 (11.3)	9 (18)	7 (14)	1 (2)	33 (66)
Neurology	5 (1.1)	2 (40)	2 (40)	1 (20)	0 (0)
Oncology	10 (2.3)	0 (0)	1 (10)	1 (10)	8 (80)
Central triage	33 (7.4)	9 (27.3)	10 (30.3)	14 (42.4)	0 (0)
ICU	27 (6.1)	2 (7.4)	2 (7.4)	2 (7.4)	21 (77.8)
Others	1 (0.2)	1 (100)	0(0)	0 (0)	0 (0)

**Figure 3:**
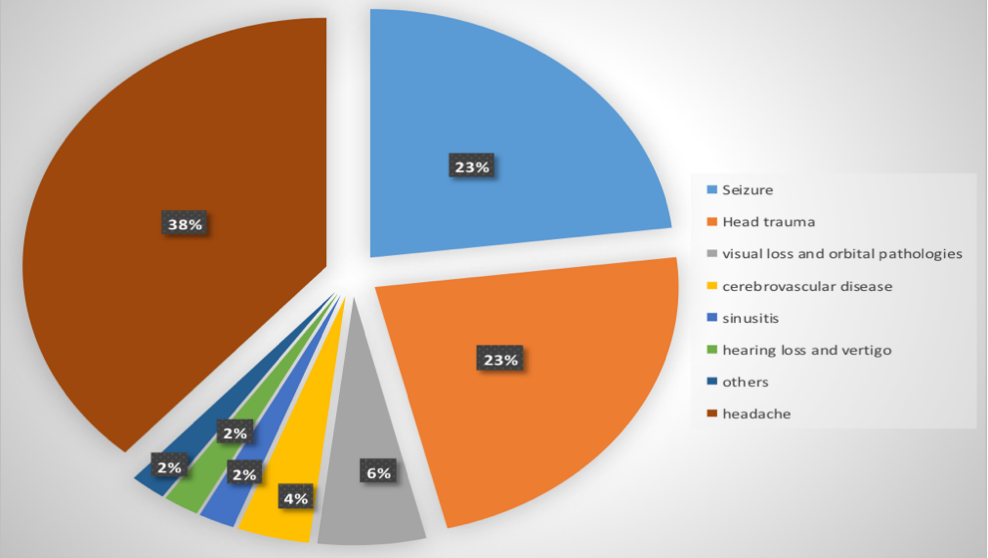
Clinical indications for Inappropriate Head CT scans

Headache (38.5%), Seizure (23.1%) and head trauma (23.1%) were the commonest indications for inappropriate scan (Figure 3). IV contrast agent was used in 80.8% of the inappropriate scans. There was no previous imaging in 84.6% of the inappropriate scans.

Residents requested 68.2% of the inappropriate scans whereas, only 6.6% of scans requested by interns were found to be inappropriate.

About 61% of the inappropriate scans were done on an elective basis and these were not pre-evaluated by radiology resident/ radiologist. Most of the inappropriate scans had either a normal outcome or just an incidental finding not related to the clinical indication (53.1% and 12.2%, respectively). Diagnostic hypothesis was confirmed in only 30.6% of the inappropriate scans.

There was statistically significant association between inappropriate scans and young age (Under 14yrs) [ AOR= 94.431 CI= 11.748–5102.402, p=0.04] and use of IV contrast agent [ AOR= 772.673, CI = 165.219–9154.197, P <0.0001].

Head CT scans done for cerebrovascular diseases were 240 more likely to be appropriate [AOR= 0.004, CI= 6.708–0.264, p= 0.01]. Interns were 4 times more likely to request an appropriate scan compared to residents [ AOR= 0.231, CI= 0.059–0.902, p= 0.035]. The odds of getting only an incidental finding is 50 times higher for inappropriate head CT scans than appropriate scans [AOR=52.086, CI= 1.577–1720.42 p= 0.027] (Table 3).

**Table 3:** Multiple logistic regression analysis results examining inappropriateness of head CT according to several variables

Independent variable		P-value	AOR	95% CI
Age	<=14	0.025***	94.43	1.75–5102.4
	15–24	0.121	25.37	0.42–1518.11
	25–34	0.048***	51.53	1.04–2545.2
	35–44	0.103	27.04	0.51–1423.33
	45–54	0.079	43.41	0.65–2905.4
	55–64	0.026***	102.67	1.72–6116.41
	≥65	1.0	1.0	1.0
Use of Contrast	Yes	0.000***	772.67	65.22–9154.2
	No	1.0	1.0	1.0
Qualification of physician	Intern	0.035***	0.23	0.06–0.9
	Resident	1.0	1.0	1.0
Mode of scan	Emergency	0.8	0.79	0.13–4.83
	Elective	1.0	1.0	1.0
Pre-evaluation by radiology resident	Yes	0.127	0.3	0.07–1.41
	No	1.0	1.0	1.0
Outcome of scan	Normal	0.07	15	0.75–302.08
	Abnormal, affecting management	0.53	2.55	0.14–47.39
	Incidental	0.03***	52.09	1.58–1720.42
	Abnormal as expected (no new finding)	1.0	1.0	1.0
Indication	Head trauma	0.12	0.09	
	Acute mental status change	0.99	2.35	
	Cerebrovascular disease	0.01***	0.004	6.7–0.26
	Headache	0.05	0.06	0.004–1.03
	Seizure	0.05	0.02	0.00–0.76
	Post craniotomy control	-	10.43	10.43–10.43
	Others	1.0	1.0	1.0
Confirmation of diagnostic hypothesis	No	0.13	7.57	0.55–104.47
	Yes	1.0	1.0	1.0

*** P< 0.05, reference category for the independent variable, the reference category for the dependent variable is “usually appropriate”

## Discussion

To the best of our knowledge, this study represents the first attempt to assess the appropriateness of head CT done at TASH. We found a significant number of inappropriate scans which was lower as compared to previous studies done in Europe and USA (1, 18, 19) and higher than studies done in South Africa, Italy and Australia (16, 20, 21).

However, comparisons with previous studies should made with caution, considering differences in methodology. In contrast to our study, most previous studies evaluated the appropriateness of imagings' of all body systems including abdominal, musculoskeletal and vascular systems (1, 20–23). In addition, we included both outpatient and inpatient examination, contrary to previous studies (1, 19, 21, 23). Moreover, in other studies, reference criteria were based on different guidelines or the ACR-AC in combination with other guidelines (19, 20, 22).

One of the commonest indications for inappropriate head CT is Headache; specifically, pre and post-contrast head CT done for Chronic Headache with no new features or neurologic deficit and new headache with red flag signs. The other common indication for inappropriate head CT was seizure. Pre and Post contrast head CT done for pediatric simple and complex febrile seizures and for first generalized seizure in neurologically normal or abnormal child were found to be inappropriate. It was also common to request non-contrast head CT scan for mild head trauma which is not indicated by either the New Orleans criteria (NOC), Canadian CT Head Rule (CCHR) or National Emergency X-ray Utilization Study (NEXUS-II) clinical criteria. This result partly matches previous studies in south Africa (6.4% inappropriate scans) among which chronic headache is the commonest indication (3, 20). The ACR-AC recommends either non-contrast head CT, non-contrast MRI or pre and post-contrast MRI for new or progressively worsening headache with ‘red flags' signs. It doesn't recommend any imaging for chronic headache with no neurologic deficit or new feature. Head MRI with and without contrast is the appropriate imaging modality for complex febrile seizures, first generalized seizure or GTC with neurologic abnormality in a child. Finally, the ACR doesn't recommend imaging for mild head trauma which is not indicated by either the NOC or CCHR or NEXUS-II clinical criteria (15).

A large number of patients imaged were aged < 14years (17.2%) and these scans were more likely to be inappropriate than the age group >65, which correlates favorably with prior studies(20, 22). Becker et. al mentioned a large number of inappropriate scans from pediatric department, similar to our result which showed 11.9% of scans from pediatric department to be inappropriate (20). Central triage, adult EOPD, and internal medicine departments requested the largest number of inappropriate scans. This can be explained by the diffuse symptoms these patients present with, leading to difficulty of the physician in choosing the right line of investigations. Other factors could include high patient load in these departments, limiting the available time to do thorough physical examination and the tendency to rely on imaging to direct patients to the appropriate specialty clinic. Vilar et.al also suggested more inappropriate scans are requested from general practice clinics than specialty clinics (22).

The qualification of requesting physician showed correlation with inappropriateness of scans. Interns were less likely to request inappropriate examination than residents in both this study an prior studies (1, 20). Possible explanations for this can be the fact that interns do not decide autonomously on most requests, they have a recent recollection of radiology course they received a year back, and partly due to the cases requiring attention by residents being more complicated ones.

Repeat scans were not frequent and mostly appropriate. This might be because prior imaging eliminates the uncertainty in localizing the patient's symptoms and helping the clinician in choosing the correct line of diagnostic investigations. This is a good practice as repeated imaging exposes patients to unnecessary radiation as well as cost (1, 2, 24–26).

Use of IV contrast correlated with inappropriateness of scan in this study and prior studies (1), in contrast to a study by Lehnert et.al which showed the highest percentage of inappropriate CT scans to be found for head CT without contrast (62%) (18). Although contrast media are essential in providing accurate diagnosis and are generally considered safe, there is a growing concern regarding associated reactions ranging from mild anaphylactic to life threatening complication (11). Inappropriate use of contrast media also subjects patients to additional cost and unnecessary inconveniences whenever contrast media are out of stock in the market.

We found association between appropriateness of imaging and confirmation of the diagnostic hypothesis (1,18). This observation validates the value of evidence-based guidelines in avoiding unnecessary procedures and orienting clinicians towards their diagnostic hypothesis (18,19).

One interesting finding in this study was that, out of the head CT requests which were pre-evaluated by radiology residents, almost 15% were found to be inappropriate. This is a significant number which should raise the question of the level of awareness of ACR-AC among radiology residents. Another explanation can be, the strictness of the ACR-AC which doesn't take into account individual patient's situation; therefore, it's possible that the radiology resident decided to do a scan, which is otherwise inappropriate according to ACR-AC, after informal communication with the requesting physician.

In the literature, several methods were shown to reduce inappropriate utilization of imaging such as manual preevaluation of requests by radiologist and computerized preauthorization programs (12,17). Preauthorization of scans needs to be properly done by radiology residents and radiologists. It should also be planned to prepare a customized appropriateness guideline.

The results of this study should be interpreted taking few potential limitations into account. First, completeness and accuracy of medical records may have distorted the actual rate of appropriateness. A significant number of requests were excluded due to missing information. Second, the ACR-AC doesn't address many clinical scenarios; for example, 12.6% of the scans in our study were done as control for patients who were status post craniotomy, which couldn't be coded with the ACR-AC. And lastly, we might have underestimated the actual percentage of appropriateness due to strict use of the ACR-AC, which does not take into account individual patient's situation including contraindications for a certain imaging modality and cost.

In conclusion, this study showed there is a significant number of inappropriately done CT scans and it should serve as a gateway for future studies to evaluate the appropriateness of all other imaging modalities done in the department. Possible associated factors such as knowledge gaps about imaging guidelines as well as availability and affordability of more appropriate imaging modalities should be identified. In addition, Medical students, physicians, radiology residents and radiologists need to be aware of the ACR appropriateness criteria and incorporate it into their daily practice in order provide better quality care for patients.
